# Supplementary material for: CEP41‐mediated ciliary tubulin glutamylation drives angiogenesis through AURKA‐dependent deciliation
Source: EMBO Rep. 2019 Dec 29;21(2):e48290. doi: 10.15252/embr.201948290 (PMC7001496; doi:10.15252/embr.201948290)
Supplement: Supplementary file 1 — Appendix [file EMBR-21-e48290-s001.pdf]

CEP41-mediated ciliary tubulin glutamylation drives angiogenesis through AURKA-dependent deciliation

Soo Mi Ki[1,8], Ji Hyun Kim[1,8], So Yeon Won[1,8], Shin Ji Oh[1], In Young Lee[2], Young-Ki Bae[3], Ki Wha Chung[4], Byung-Ok Choi[5], Boyoun Park[6], Eui-Ju Choi[2], & Ji Eun Lee[1,7]

Table of contents **Page**

Table of contents	Page
Appendix Table S1	2
Appendix Table S2	3
Appendix Table S3	4
Appendix Figure S1	5
Appendix Figure S2	6
Appendix Figure S3	8
Appendix Figure S4	9
Appendix Figure S5	10
Appendix Figure S6	11
Appendix Figure S7	12
Appendix Figure S8	13
Appendix Figure S9	14

Appendix Table S1. Sequences of siRNAs used in this study

**Gene**		**Sequence**
CEP41 siRNA#1		5’-CUGCUAGAUGUGCGUGAUA-3’
CEP41 siRNA#2		5’-GGUAACUGCUACGGGGUAA-3’
HIF1α siRNA #1		5’-CUAACUGGACACAGUGUGU-3’
HIF1α siRNA #2		5’-ACACACUGUGUCCAGUUAG-3’
CCP5 siRNA pool	siRNA #1	5’-GUGUAGAUCUGCUGACGAU-3’
	siRNA #2	5’-GAGUGGCGUUGCUUAC-3’
	siRNA #3	5’-GAAUCUUUGUCCAGUGAUG-3’
	siRNA #4	5’-UGACAGGCAUAACGCUGAA-3’
ON-TARGET *plus* Non-targeting pool		5’-UGGUUUACAUGUCGACUAA-3’
		5’-UGGUUUACAUGUUGUGUGA-3’
		5’-UGGUUUACAUGUUUUCUGA-3’
		5’-UGGUUUACAUGUUUUCCUA-3’

Appendix Table S2. Primer sequences for quantitative Real Time-PCR

Gene	Forward sequence	Reverse sequence
Human *CEP41*	5-TTCTGTATGACGATGATGAAAGG-3’	5’-TTCCGGGAATTTCTGAGCTA-3’
Human *CCP5*	5’-CTCCCCGGGTCTGATAATGC-3’	5’-ACGGCTGTCACATGGAATCA-3’
Human *VEGFA*	5’-CAGTTCGAGGAAAGGGAAAGG-3’	5’-CAACGCGAGTCTGTGTTTTTG-3’
Human *VEGFR2*	5’-GCAATCCCTGTGGATCTGAA-3’	5’-ACTCCATGCCCTTAGCCACT-3’
Human *HIF1α*	5’-CATAAAGTCTGCAACATGGAAGGT-3’	5’-ATTTGATGGGTGAGGAATGGGTT-3’
Human *GAPDH*	5’-TGACTTCAACAGCGACACCCA-3’	5’-CACCCTGTTGCTGTAGCCAAA-3’
Zebrafish *vegfa*	5’-GAAAACCACTGTGAGCCTTG-3’	5’-GCAGGAGCATTTACAGGTGA-3’
Zebrafish *vegfr2*	5’-TACAGACCCGGCCAAACAA-3’	5’-TTTCCCCCCTGGAAATCCT-3’
Zebrafish *β actin*	5’-GATCTTCACTCCCCTTGTTC-3’	5’-ATACCGGAGCCGTTGTCA-3’

Appendix Table S3. Sequences of morpholinos used in this study

Gene	Morpholino type	Sequence
cep41	translating blocking (AUG)	5’-CATCTTCCAGCAGCAGAGCTTCGGC-3’
cep41	splicing blocking (SB)	5’-AGCTGGTAAGAAGAAGAGTATATTA-3’
ccp5	translating blocking (AUG)	5’-TCCTCTTAATGTGCAGATACCCGTT-3’

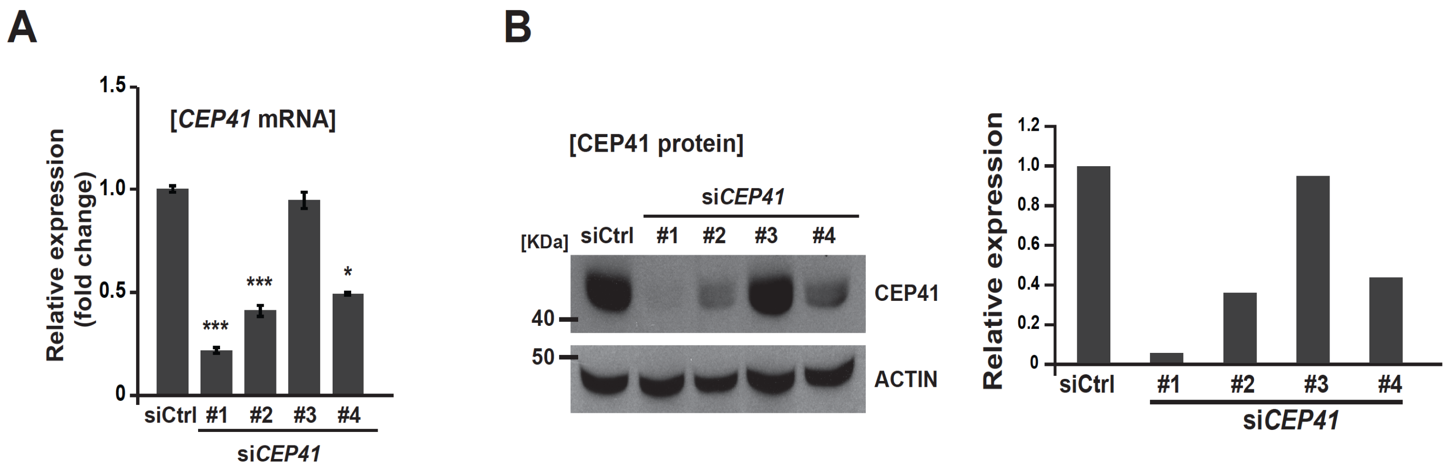

Appendix Figure S1. The knockdown efficiency tests of *CEP41* siRNAs. **A**, HUVECs were transfected with four individual *CEP41* siRNAs and collected for qRT-PCR for a *CEP41* knockdown efficiency test. The results revealed that the #1 and #2 siRNAs depleted *CEP41* most efficiently. Thus, these *CEP41* siRNAs were selected for the knockdown experiments in this study. **B**, The *CEP41* siRNAs-transfected HUVECs were collected for immunoblot assay to examine protein level of CEP41. The cells transfected with the #1 and #2 siRNAs showed the lowest abundance of CEP41 protein, which was consistent with the result of qRT-PCR results shown in (**A**). The data are shown as the mean ± SD: $*P < 0.05$, $***P < 0.001$ (Student's *t*-test).

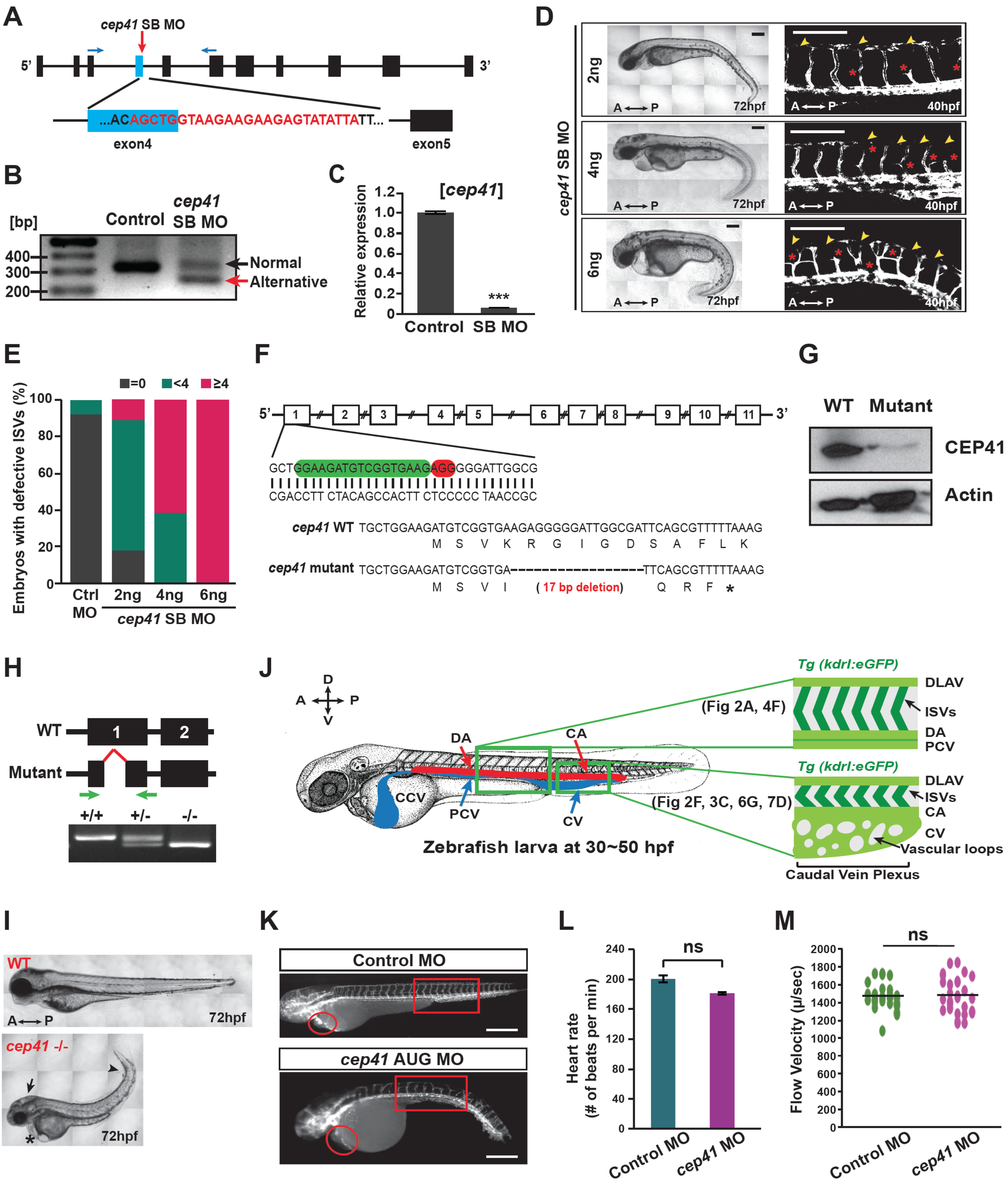

A
cep41 SB MO
5'
3'
...ACAGCTGGTAAGAAGAAGAGTATATTATT...
exon4
exon5
B
[bp]
Control
cep41 SB MO
400
300
200
Normal
Alternative
C
[cep41]
Relative expression
1.2
1.0
0.8
0.6
0.4
0.2
0
***
Control
SB MO
D
cep41 SB MO
2ng
4ng
6ng
A ⟷ P
72hpf
40hpf
E
=0
<4
≥4
Embryos with defective ISVs (%)
100
80
60
40
20
0
Ctrl MO
2ng
4ng
6ng
cep41 SB MO
F
5'
1
2
3
4
5
6
7
8
9
10
11
3'
GCTGGAAGATGTCGGTGAAGAGGGGGATTGGCG
CGACCTT CTACAGCCACTT CTCCCCC TAACCGC
cep41 WT TGCTGGAAGATGTCGGTGAAGAGGGGGATTGGCGATTCAGCGTTTTTAAAG
M S V K R G I G D S A F L K
cep41 mutant TGCTGGAAGATGTCGGTGA-----------------TTCAGCGTTTTTAAAG
M S V I (17 bp deletion) Q R F *
G
WT
Mutant
CEP41
Actin
H
WT
Mutant
1
2
+/+
+/-
-/-
J
D
A
P
V
DA
CA
CCV
PCV
CV
Zebrafish larva at 30~50 hpf
Tg (kdrl:eGFP)
(Fig 2A, 4F)
DLAV
ISVs
DA
PCV
(Fig 2F, 3C, 6G, 7D)
CA
CV
Vascular loops
Caudal Vein Plexus
I
WT
A ⟷ P
72hpf
cep41 -/-
*
72hpf
K
Control MO
cep41 AUG MO
L
ns
240
200
160
120
80
40
0
Heart rate
(# of beats per min)
Control MO
cep41 MO
M
ns
2000
1800
1600
1400
1200
1000
800
600
400
200
0
Flow Velocity (μl/sec)
Control MO
cep41 MO

Appendix Figure S2. Observations of vascular system in the *cep41*-depleted zebrafish. A, A schematic displaying the location of *cep41* splice-blocking (SB) morpholino (MO) targeting the junction between exon 4 and intron 4. **B**, The result of RT-PCR using the *cep41* SB MOs-injected zebrafish embryos reveals the abnormal *cep41* product generated by alternative splicing (a red arrow). **C**, The result of qRT-PCR reveals the reduced *cep41* product in SB MOs-injected zebrafish. ***$P < 0.005$ (Student's *t*-test). **D**, The *kdrl:eGFP* transgenic zebrafish were injected with *cep41* MOs (2~6ng/nl) and analyzed to observe the dosage-dependent defects in zebrafish vasculature at 40 hours post-fertilization (hpf). Asterisks and arrowheads indicate the malformed ISVs and DLAVs, respectively. Scale bars, 200 µm. **E**, The quantified data of morphants with defective ISVs within eight somites were displayed graphically. **F**, Top: A CRISPR guide was designed to target the first exon of *cep41*. The target sequence is highlighted in green and the PAM sequence in red. Bottom: Sequencing results of the *cep41* locus in the wild type and mutant fish. An asterisk indicates the pre-generated stop codon. **G**, Western blotting results indicating the ablation of cep41 protein in the *cep41* mutant embryos. **H**, Top: Location of the 17 bp deletion and PCR primers used for genotyping. Bottom: Genotyping results using the primers. **I**, Morphological phenotypes of the *cep41* knock-out zebrafish at 72 hpf. An arrow, an arrowhead, and an asterisk indicate hydrocephalus, heart edema, and curved tail, respectively as representative ciliopathy defects. **J**, A diagram represents zebrafish vasculature by indicating the areas shown in main Figures. A, anterior; P, posterior; D, dorsal; V, ventral; DA, dorsal artery; CA, caudal artery; CCV, common cardinal vein; PCV, posterior cardinal vein; CV, caudal vein; DLAV, dorsal longitudinal anastomotic vessel; ISVs, intersegmental vessels. **K**, The *Tg(kdrl:eGFP)* zebrafish, injected with control or *cep41* MOs, were used for recording to analyze heart beating (Movie. S1 and S2) and blood flow velocity (Movie. S3 and S4) at 48 hpf. Ovals and rectangles indicate the analyzed areas of hearts and blood vessels, respectively. Scale bars, 500 µm. **L**, The heart rate measured in each morphants is presented graphically. **M**, The graph displays the flow velocity analyzed in the morphants. The data are shown as the mean ± SD. (Unpaired *t*-test with Welch's correction). Over 20 zebrafish embryos were analyzed for each experiment.

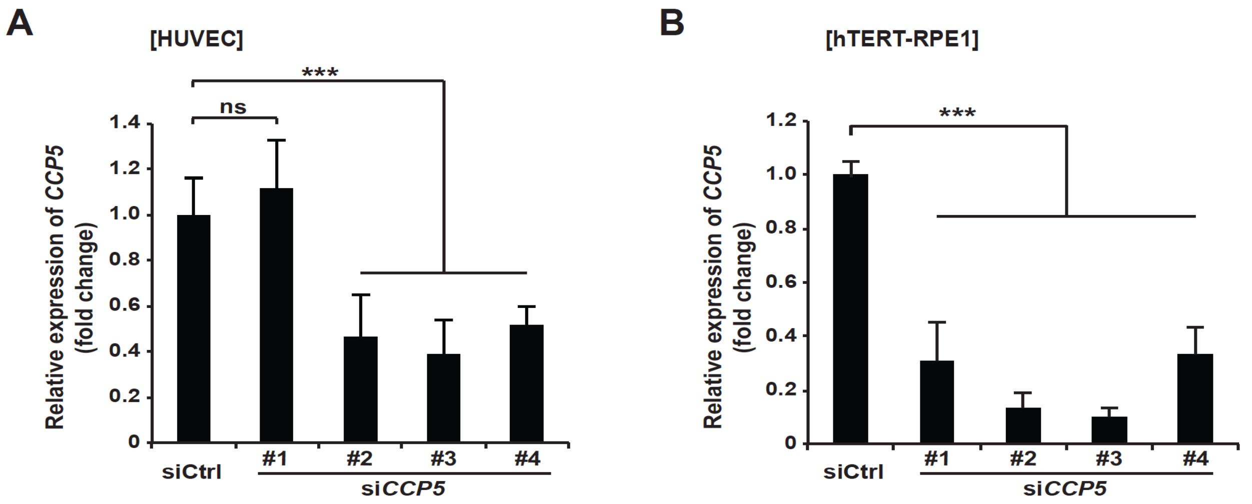

Appendix Figure S3. The knockdown efficiency tests of *CCP5* siRNAs. A and **B**, HUVECs (**A**) and hTERT- RPE1 (**B**) were transfected with four individual *CCP5* siRNAs and collected for qRT-PCR for a *CCP5* knockdown efficiency test. The #2 and #3 siRNAs that efficiently depleted *CCP5* in both of cell lines were used for this study. The data are shown as the mean ± SD: ***P < 0.001 (One-way ANOVA by Dunnett's *post hoc* test).

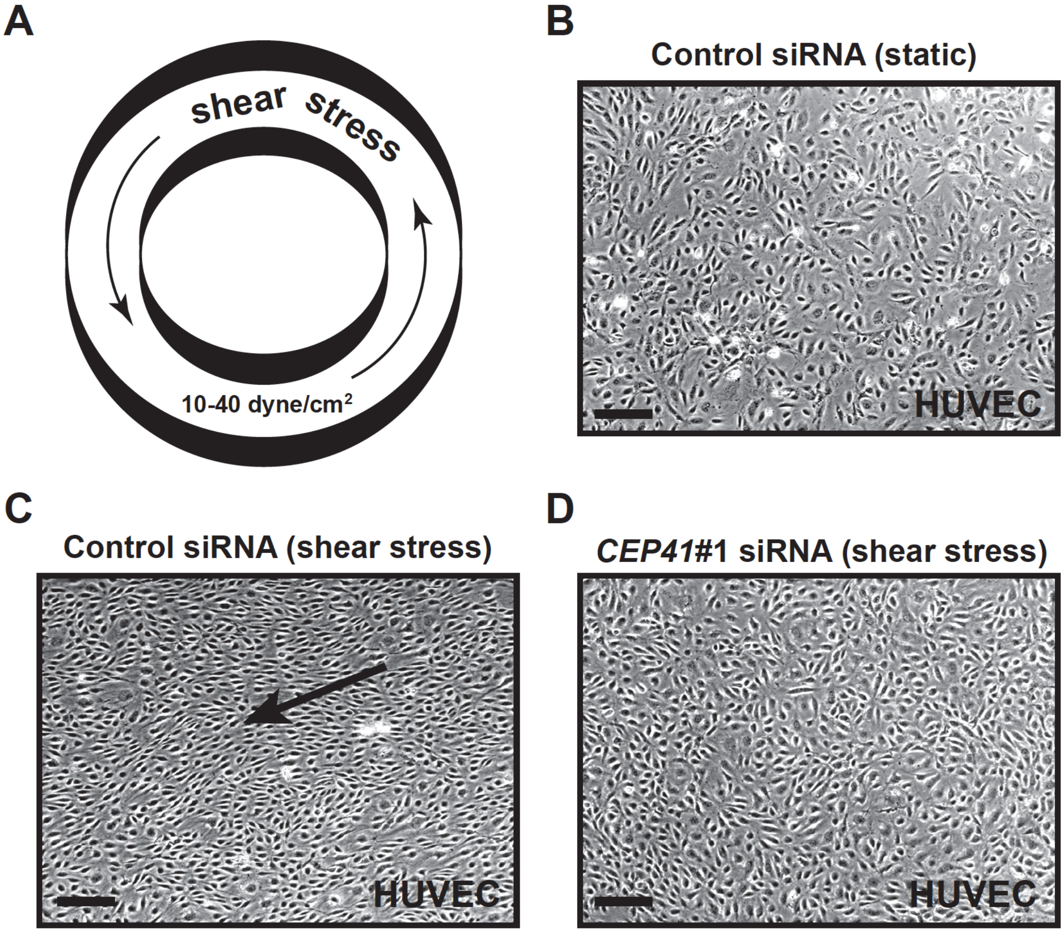

Appendix Figure S4. Depletion of *CEP41* prevents sensing of laminar shear stress of HUVECs. A, A diagram displays a mode to induce flow-mediated shear stress (10-40 dyne/cm^2) using cell culture dishes. Cells are plated on the peripheral area indicated with arrows, which present the direction of flow-driven shear stress. **B**, Control siRNA-transfected HUVECs under static state show non-directional cell populations. **C** and **D**, Under shear stress, control cells change the cell morphologies according to the direction of shear stress (an arrow), whereas *CEP41*-knockdown cells show no different morphologies. Scale bars, 600 μm.

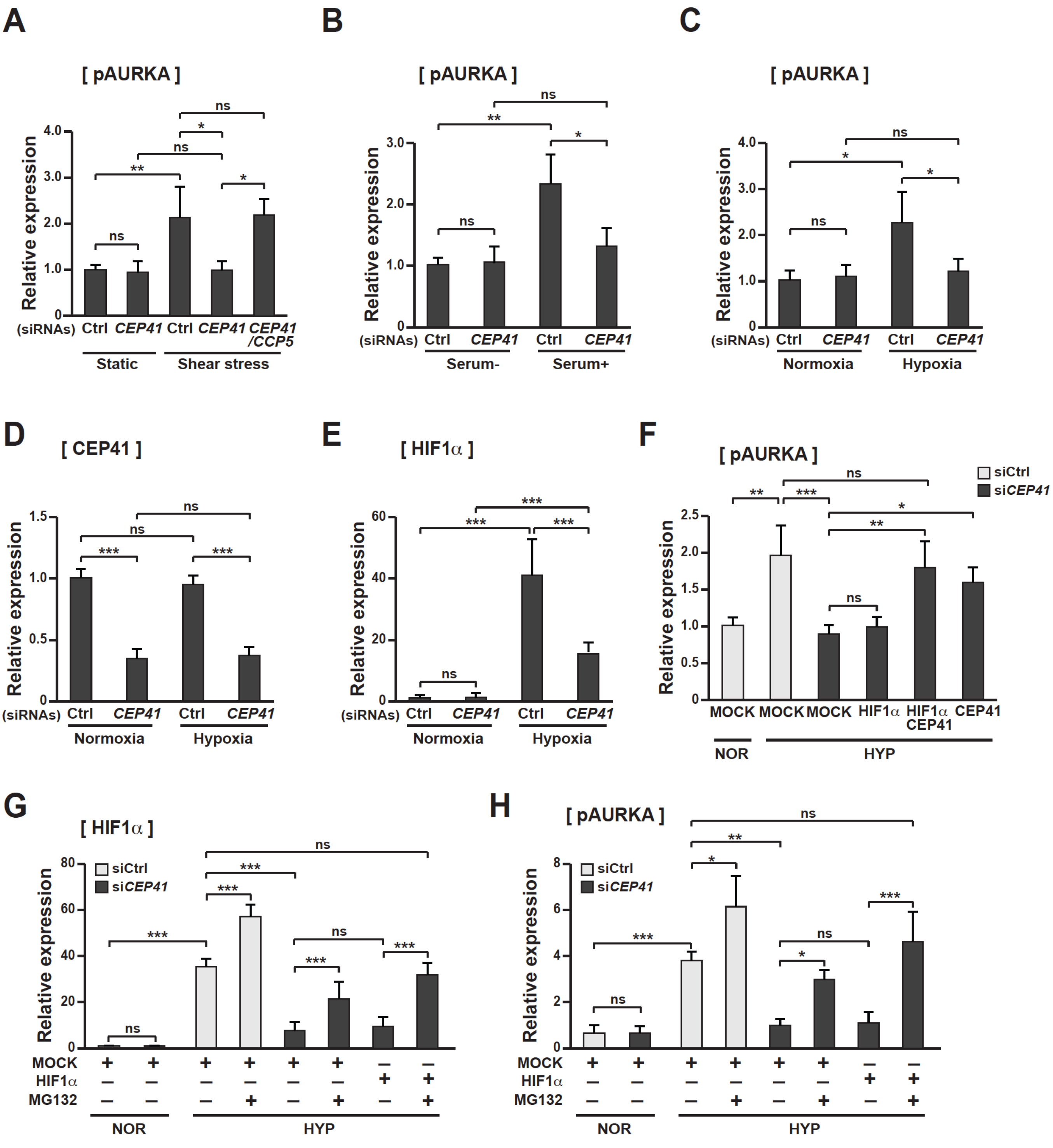

Appendix Figure S5. Quantification of protein levels from immunoblot assays. Quantified data of the representative western blots, which are presented in the main figures are shown. **A**, Quantification of the immunoblot results in Fig 6C. **B**, Quantification of the immunoblot results in Fig 6D. **C** and **D**, Quantification of the immunoblot results in Fig 7A. **E**, Quantification of the immunoblot results in Fig 8A. **F**, Quantification of the immunoblot results in Fig 8D. **G** and **H**, Quantification of the immunoblot results in Fig 8G. Data are mean ± SD of more than three independent immunoblot assays per condition. Statistical significance was determined using the one-way ANOVA followed by Tukey's *post hoc* test (*$P < 0.05$, **$P < 0.01$, ***$P < 0.001$, ns: non-significant).

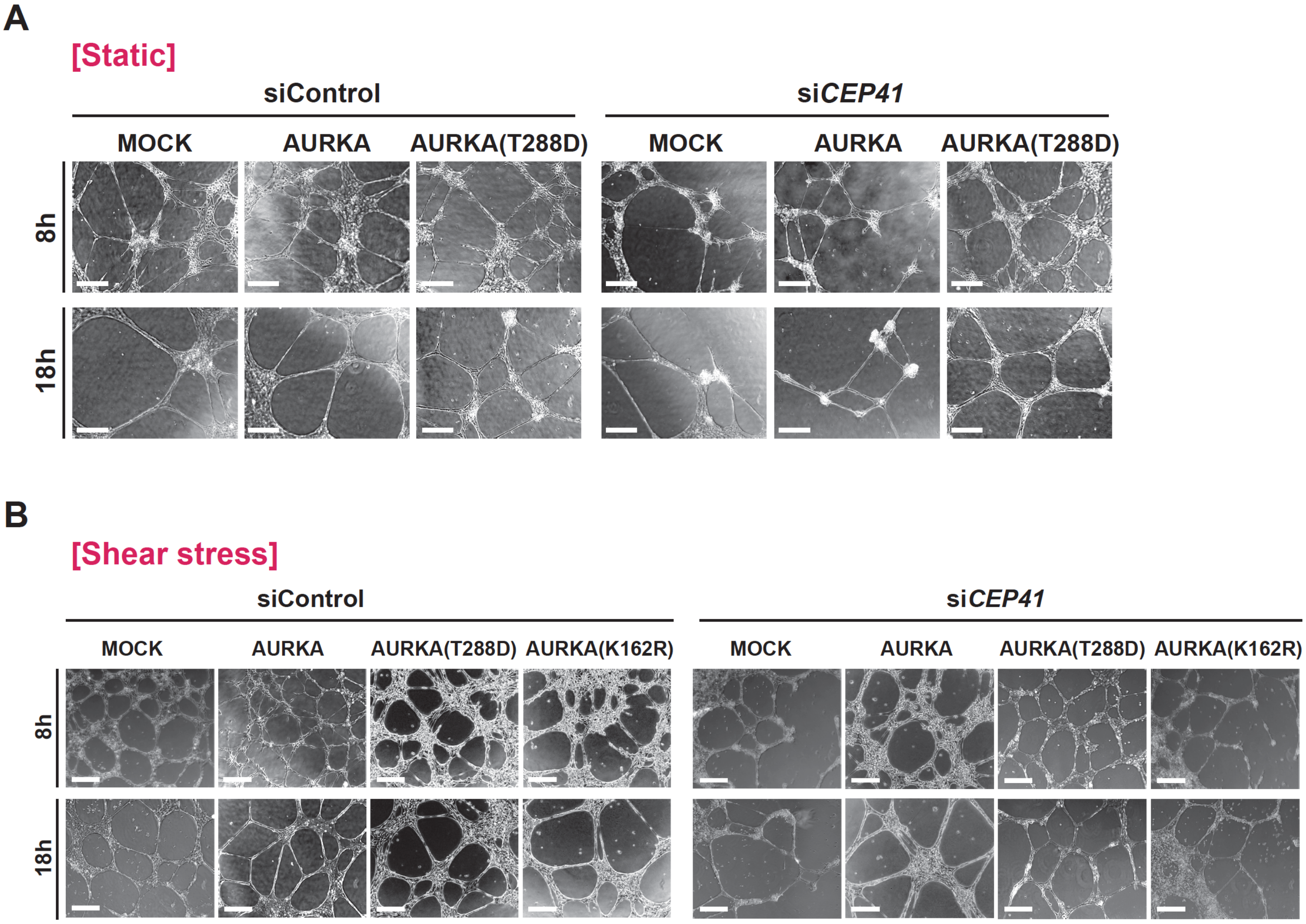

Appendix Figure S6. Activation of CEP41-mediated AURKA is responsible for tubulogenesis in HUVECs under shear stress. A and **B**, Control and *CEP41*-deficient HUVECs were transfected with expression vectors encoding nothing (MOCK), AURKA, AURKA-T288D, or AURKA-K162R and subjected to an *in vitro* angiogenesis assay for 18 h under static (**A**) or shear stress (**B**) states. Representative data are from five independent experiments with ≥ 5 tubulogenesis regions per condition. Scale bars, 600 µm.

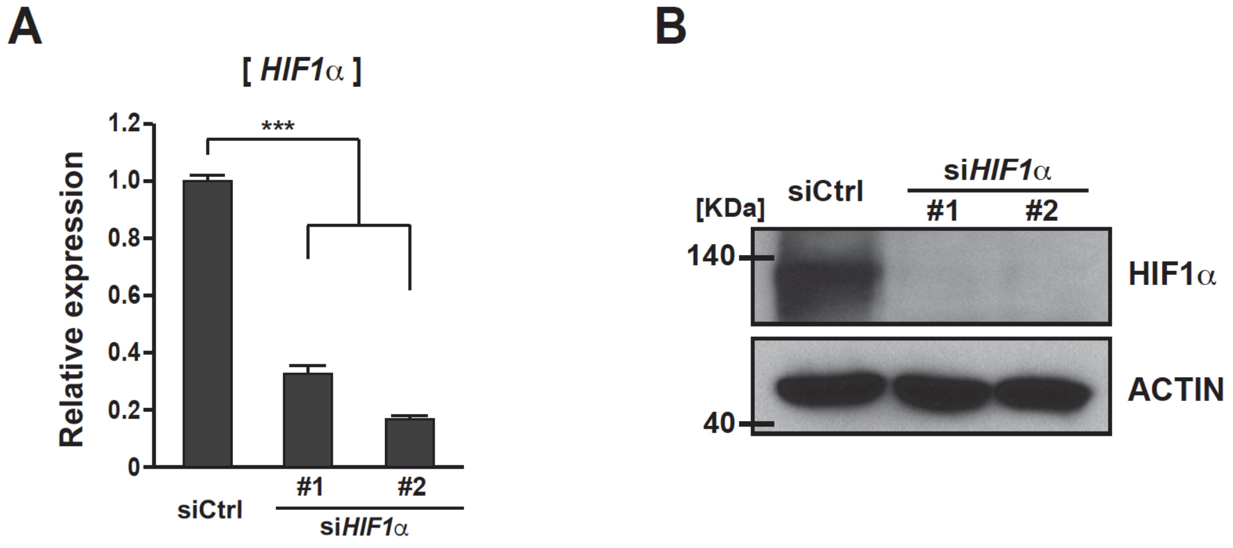

Appendix Figure S7. The knockdownnefficiency tests of *HIF1*α siRNAs. A, HUVECs were transfected with two individual *HIF1*α siRNAs and collected for qRT-PCR for a *HIF1*α knockdown efficiency test. **B**, The *HIF1*α siRNAs-transfected HUVECs were collected for immunoblot assay to examine protein level of HIF1α. The cells transfected with the #1 and #2 siRNAs showed absence of *HIF1*α protein. The data are shown as the mean ± SD: $^{***}P < 0.001$ (One-way ANOVA with Dunnett's *post hoc* test).

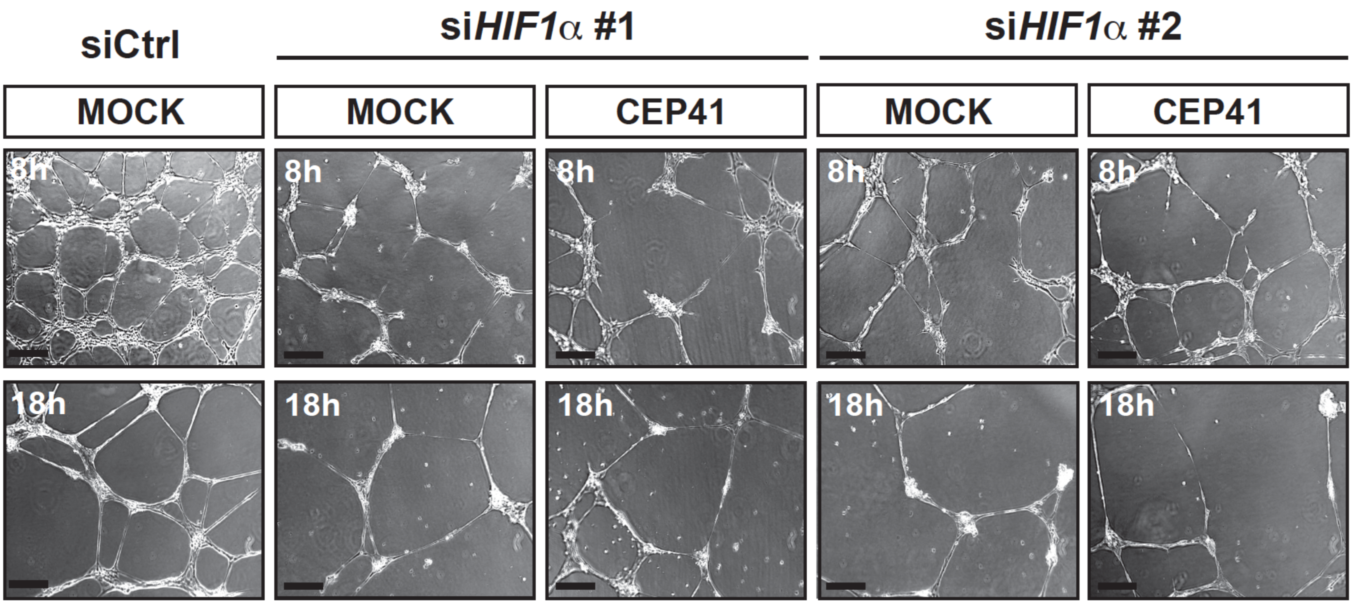

Appendix Figure S8. Exogenous CEP41 is unable to restore the angiogenic defects by *HIF1*α depletion. Control and *HIF1*α-deficient HUVECs were transfected with expression vectors encoding nothing (MOCK) or CEP41 and subjected to an *in vitro* angiogenesis assay for 18 h under hypoxia states. Representative data are from five independent experiments with ≥ 5 tubulogenesis regions per condition. Scale bars, 600 μm.

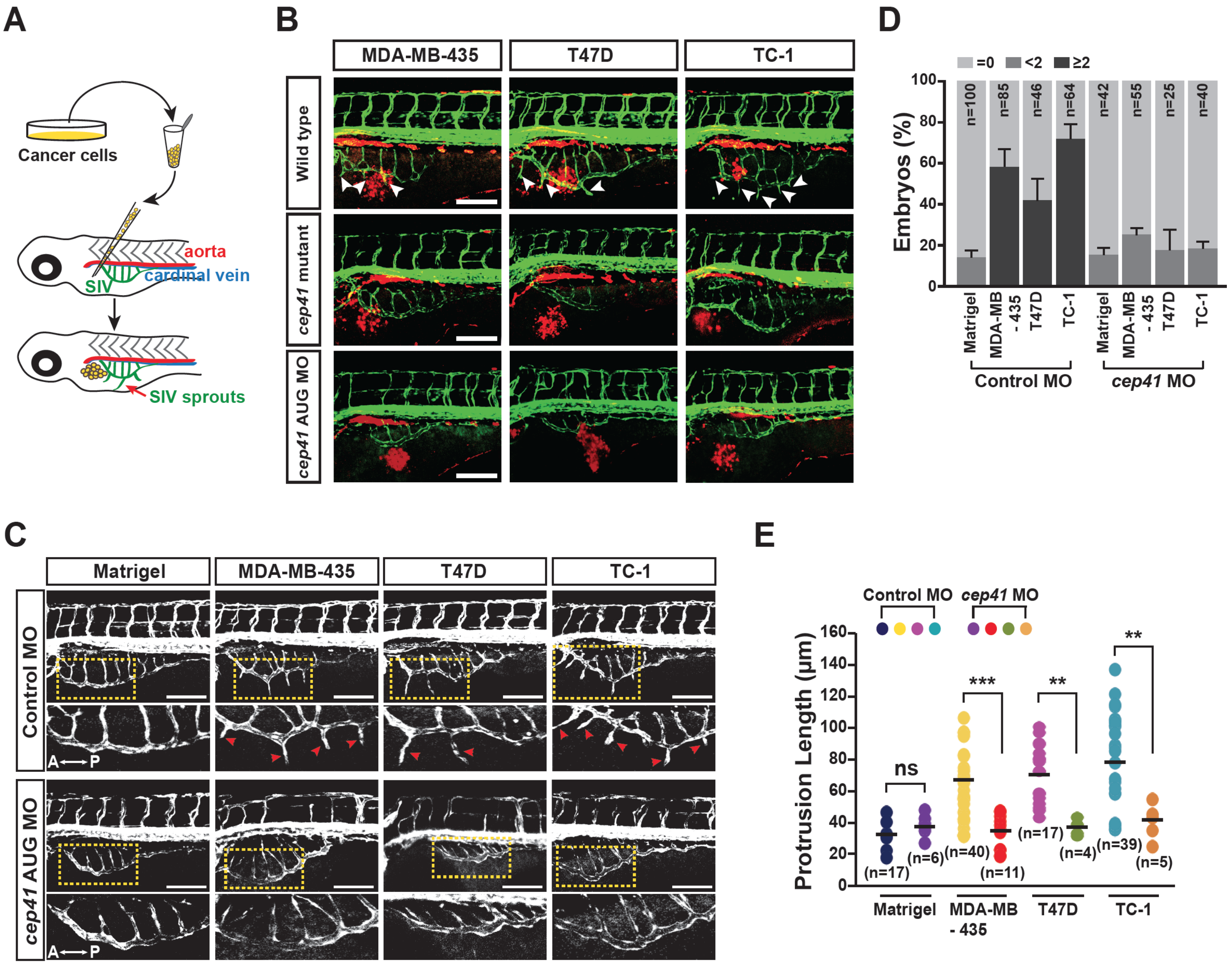

Appendix Figure S9. Ablation of *cep41* affects tumor-induced angiogenesis. A , A diagram represents the experi-mental scheme of zebrafish xenograft. Several kinds of mammalian cancer cells are cultivated in vitro and trans-planted into zebrafish larvae at 48 hpf. The tumor-induced angiogenesis was analyzed by observing the additional subintestinal vein plexus (SIV) sprouts. **B** and **C**, The MDA- MB-435 and the T47D (human breast cancer cells), and the TC-1 (mouse lung cancer cells) cells were labeled with DiI and then transplanted into *cep41* mutant or *cep41* AUG MO -injected zebrafish at 48 hpf. The additional SIV (arrowheads) were analyzed and compared to those of the wild type or control MO-injected zebrafish 24 h after xenograft. The dotted rectangles indicate the areas observed in the individual zebrafish, and the areas are presented as magnified images at the bottom. Scale bars, 200 µm. SIV, subintestinal vein plexus. **D**, The branch numbers of additional SIV were quantified and presented graphically. **E**, The length of SIV protrusions was measured in zebrafish only with additional SIV and the data were displayed in the graph. The data are shown as the mean ± SD; $^{**}P < 0.01$, $^{***}P < 0.001$, ns: non-significant (Student's *t*-test).
