# Supplementary material for: CEP41‐mediated ciliary tubulin glutamylation drives angiogenesis through AURKA‐dependent deciliation
Source: EMBO Rep. 2019 Dec 29;21(2):e48290. doi: 10.15252/embr.201948290 (PMC7001496; doi:10.15252/embr.201948290)
Supplement: Supplementary file 2 — Expanded View Figures PDF [file EMBR-21-e48290-s002.pdf]

Expanded View Figures

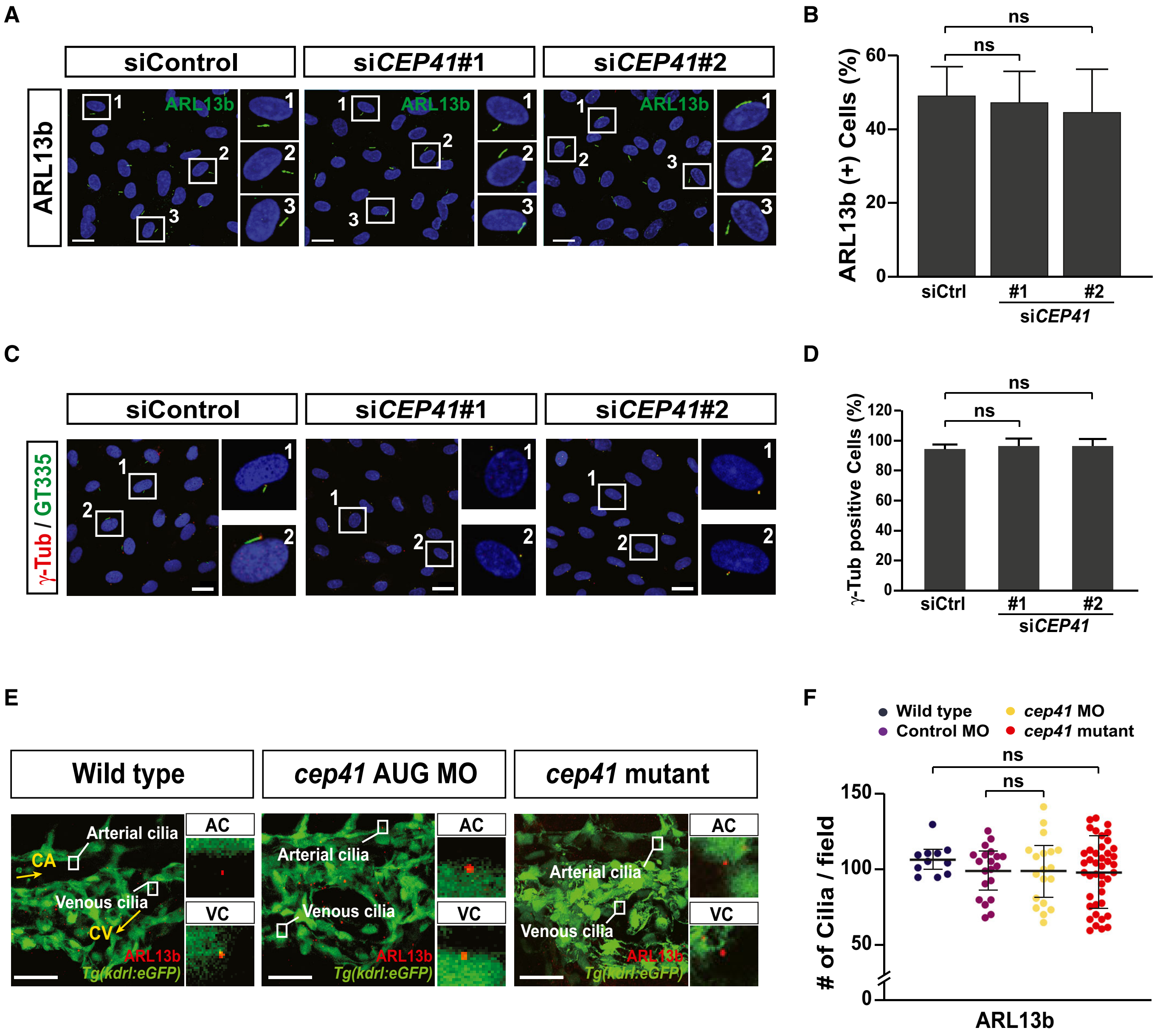

Figure EV1. CEP41 is dispensable for generation of the centrosome and the cilium.

A–D Control or *CEP41* siRNA-transfected HUVECs were immunostained with ARL13b or γ-Tub-specific antibodies in (A, C). The rectangles indicate the representative cells from each immunostaining experiment presented as magnified images in the right panels. Scale bars, 20 μm. Quantification of the ciliated cell numbers in the images in (B, D) from the results of three independent experiments with ≥ 200 cells per condition (mean ± SD). ns: non-significant (one-way ANOVA with Tukey's *post hoc* test).

E, F Wild-type and *cep41*-deficient *Tg(kdrl:eGFP)* zebrafish embryos were fixed for immunostaining with ARL13b-specific antibodies at 28 hpf in (E). The rectangles indicate the arterial cilia (AC) and venous cilia (VC) in zebrafish endothelial cells. Magnified representative images are displayed in the right panels. CA, caudal artery; CV, caudal vein. Scale bars, 40 μm. Quantification of the labeled cilia observed in equivalent fields of view is presented graphically in (F). Data are shown as mean ± SD of three independent experiments (*n* ≥ 20 embryos per condition). Statistical significance was determined using the one-way ANOVA followed by Tukey's *post hoc* test (ns: non-significant).

© 2019 The Authors

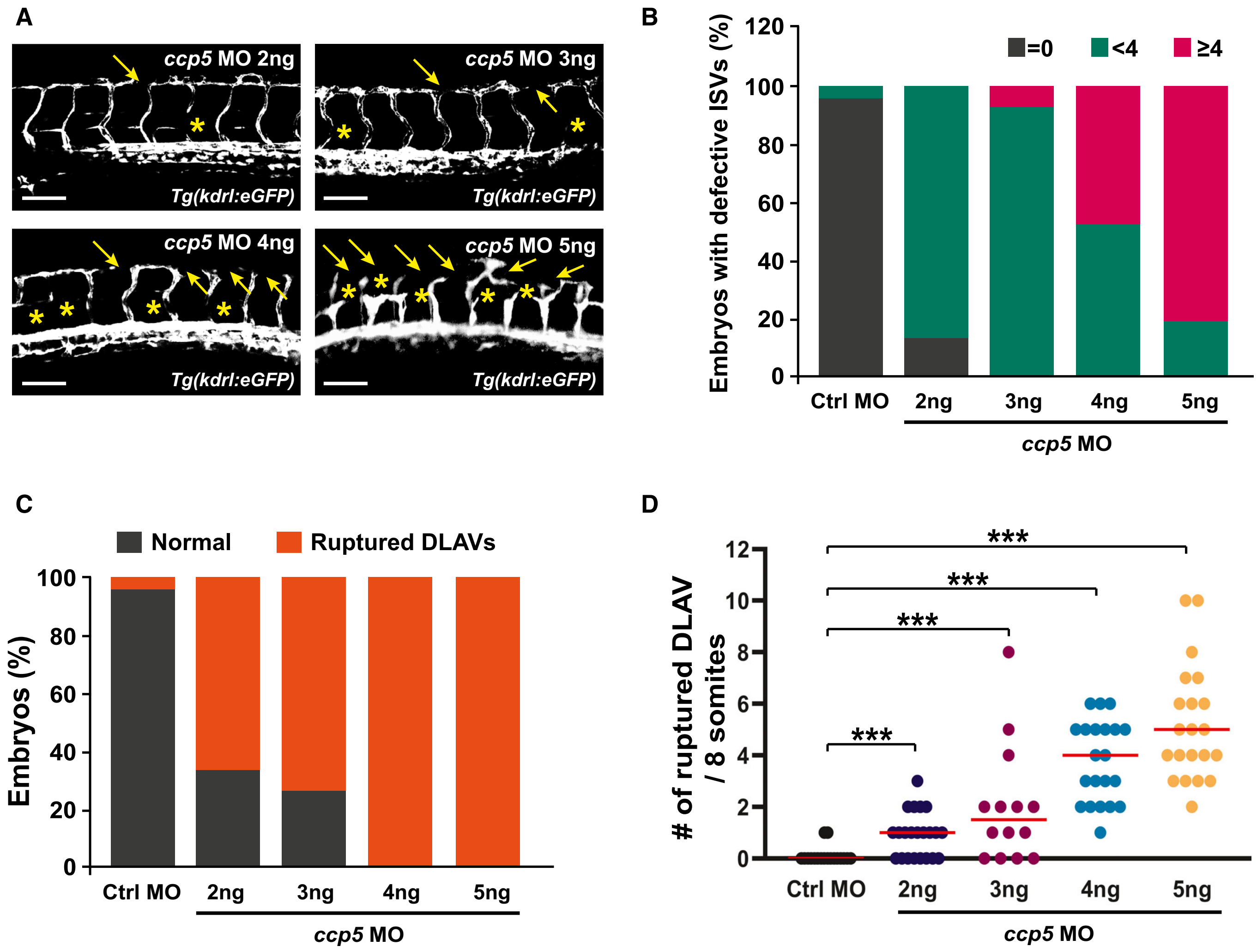

Figure EV2. Depletion of ccp5 leads to defects in the sprouting angiogenesis.

A Blood vessels were observed in *ccp5* AUG MOs (2–5 ng/nl)-injected *Tg(kdrl:eGFP)* zebrafish at 40 hpf by fluorescent microscopy. Asterisks and arrowheads indicate impaired ISVs and DLAVs, respectively. Scale bars, 100 μm.

B–D Quantification of the numbers of embryos with defective ISVs in (B), the numbers of embryos with aberrant DLAVs in (C), and the numbers of ruptured DLAVs in (D) from data observed in equivalent fields of view (within eight somites). Data are shown as median of three independent experiments with ≥ 20 embryos per condition. Statistical significance was determined using the Brown–Forsythe ANOVA followed by Dunnett's T3 *post hoc* test (***$P < 0.001$).

 © 2019 The Authors

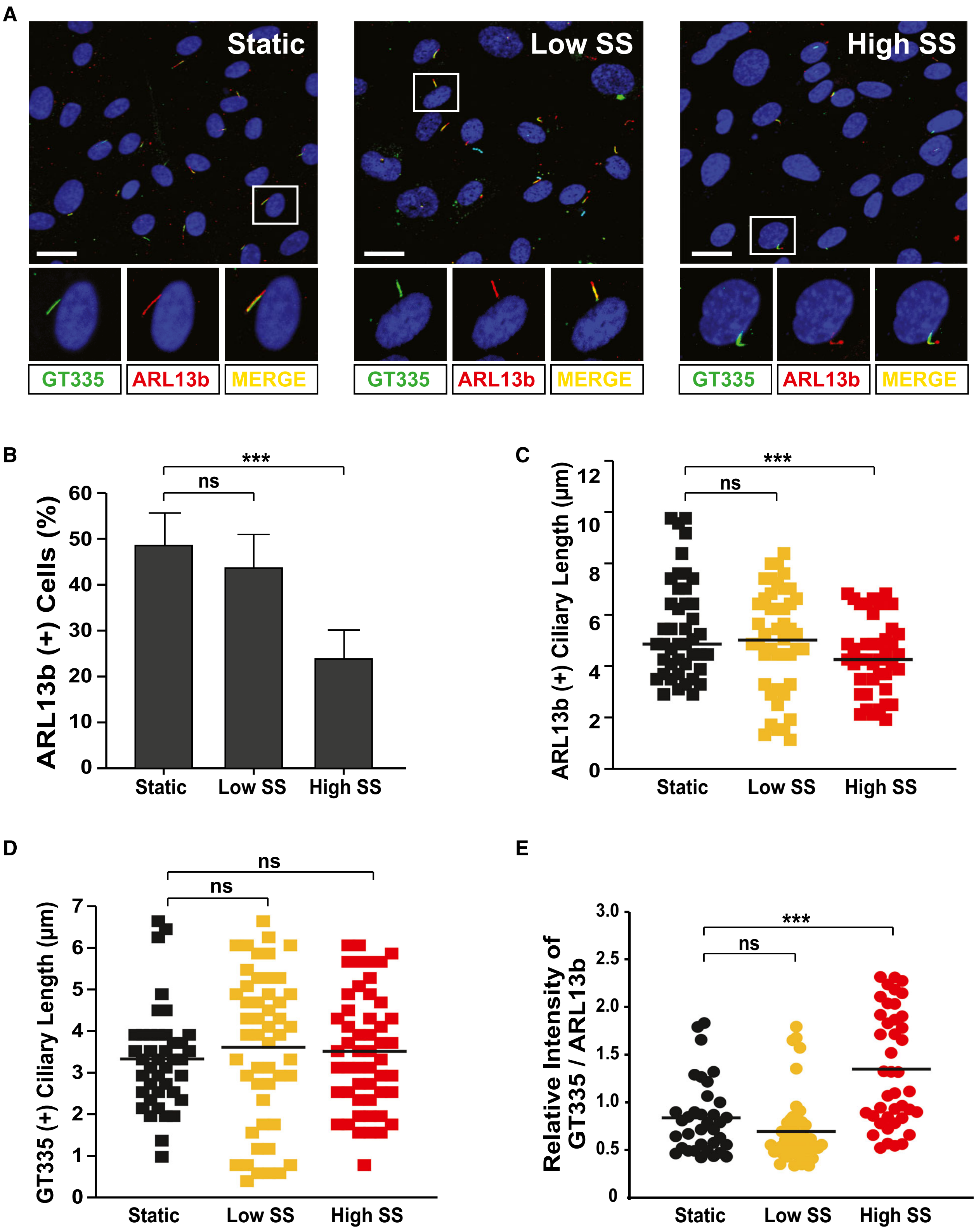
A
Static
Low SS
High SS
GT335
ARL13b
MERGE
B
ARL13b (+) Cells (%)
ns
***
Static
Low SS
High SS
C
ARL13b (+) Ciliary Length (μm)
ns
***
D
GT335 (+) Ciliary Length (μm)
ns
E
Relative Intensity of GT335 / ARL13b

Figure EV3.

Figure EV3. High shear stress induces ciliary tubulin glutamylation and cilia disassembly in HUVECs.

A HUVECs were cultivated under either static, low, or high shear stress conditions and were immunostained with ARL13b- and GT335-specific antibodies. Representative images indicated with rectangles appear in magnified images in the lower panels. Scale bars, 20 μm.

B–E Quantification of the numbers of ciliated cells (B), ciliary length (C, D), and relative intensity of GT335/ARL13b signals in the cilia (E) in images (A) is the result of three independent experiments with ≥ 200 cells per condition (mean ± SD or median). ***$P < 0.001$, ns: non-significant (one-way ANOVA with Dunnett's *post hoc* test (B–E)).

Figure EV4. Hypoxia induces ciliary tubulin glutamylation and cilia disassembly in HUVECs.

A HUVECs were cultivated under either normoxia or hypoxia (short and long exposure) conditions and were immunostained with ARL13b- and GT335-specific antibodies. Representative images indicated with rectangles appear in magnified images in the lower panels. Scale bars, 20 μm.

B–E Quantification of the numbers of ciliated cells (B), ciliary length (C, D), and relative intensity of GT335/ARL13b signals in the cilia (E) in images (A) is the result of three independent experiments with ≥ 200 cells per condition (mean ± SD or median). **$P < 0.001$, ***$P < 0.001$, ns: non-significant (one-way ANOVA with Dunnett's *post hoc* test (B, D) and Brown–Forsythe ANOVA with Dunnett's T3 *post hoc* test (C) and Kruskal–Wallis test with Dunn's *post hoc* test (E)).

 © 2019 The Authors

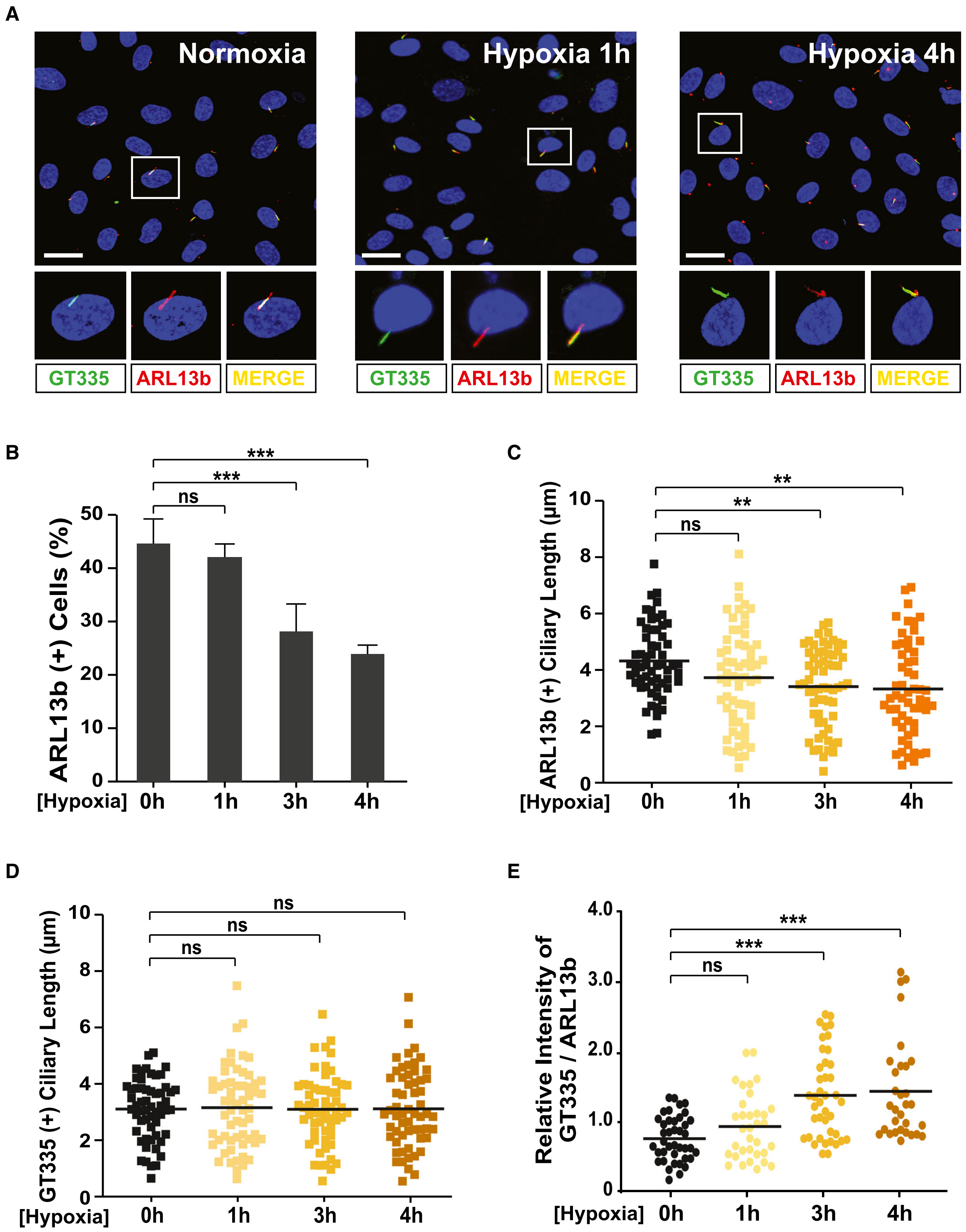
A
Normoxia
Hypoxia 1h
Hypoxia 4h
GT335
ARL13b
MERGE
B
ARL13b (+) Cells (%)
[Hypoxia] 0h 1h 3h 4h
ns
***
***
C
ARL13b (+) Ciliary Length (μm)
[Hypoxia] 0h 1h 3h 4h
ns
**
**
D
GT335 (+) Ciliary Length (μm)
[Hypoxia] 0h 1h 3h 4h
ns
ns
ns
E
Relative Intensity of GT335 / ARL13b
[Hypoxia] 0h 1h 3h 4h
ns
***
***

Figure EV4.

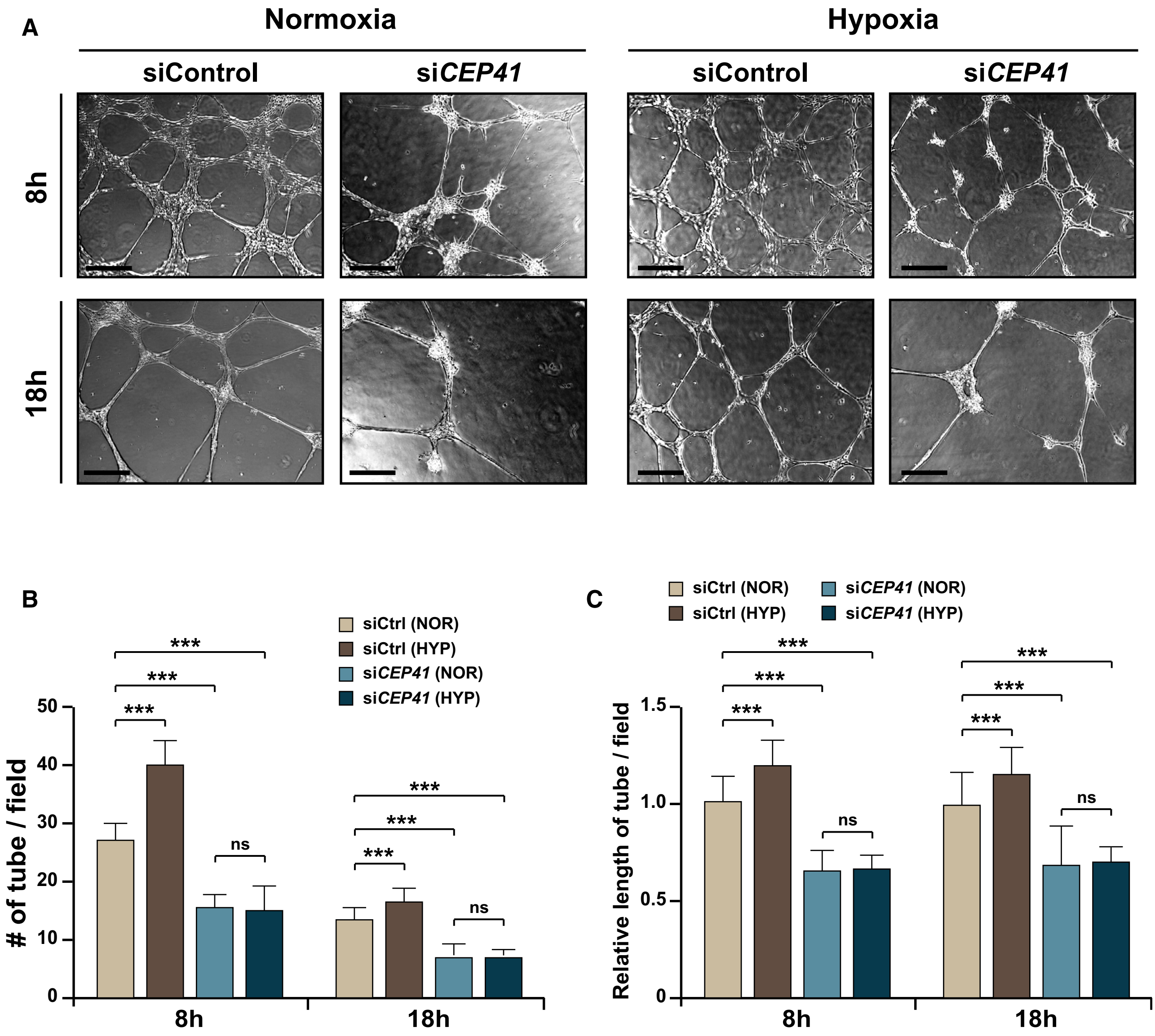

Figure EV5. Enhanced angiogenesis by hypoxia induction is inhibited by CEP41 depletion in HUVECs.

A An *in vitro* angiogenesis assay was performed in normoxia- or hypoxia-exposed HUVECs transfected with control or *CEP41* siRNAs for 18 h. Scale bars, 600 µm.

B, C Quantification of tube node number in (B) and tube length in (C) from data examined within equivalent fields of view at each time point using the ImageJ angiogenesis analyzer. Data are shown as mean ± SD of five independent experiments with ≥ 5 tubulogenesis regions per condition. Statistical significance was determined using the two-way ANOVA followed by Tukey's *post hoc* test (***$P < 0.001$, ns: non-significant).

 © 2019 The Authors
