# Supplementary material for: CEP41‐mediated ciliary tubulin glutamylation drives angiogenesis through AURKA‐dependent deciliation
Source: EMBO Rep. 2019 Dec 29;21(2):e48290. doi: 10.15252/embr.201948290 (PMC7001496; doi:10.15252/embr.201948290)
Supplement: Supplementary file 4 — Movie EV2 [file EMBR-21-e48290-s004.zip › MovieEV2/MovieEV2_Legend.docx]

**Movie EV2**

Observation of heart beating in *cep41* MOs-injected zebrafish at 48 hpf.
