# Supplementary figures and images for: CEP41‐mediated ciliary tubulin glutamylation drives angiogenesis through AURKA‐dependent deciliation
Source: EMBO Rep. 2019 Dec 29;21(2):e48290. doi: 10.15252/embr.201948290 (PMC7001496; doi:10.15252/embr.201948290)

Appendix Fig S1B

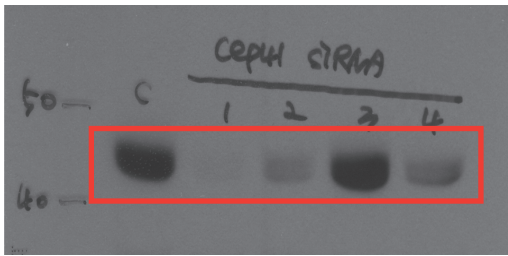

[CEP41]

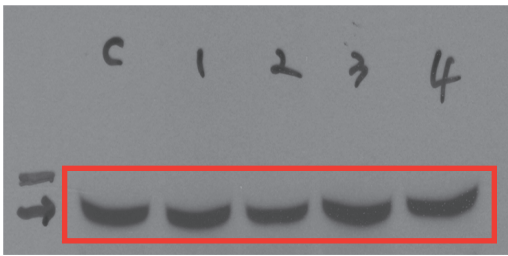

[ACTIN]

Supplement: Supplementary file 7 — Source Data for Appendix [file EMBR-21-e48290-s010.zip › Source_data_App_Fig_S1.pdf]

## Appendix Fig S2G

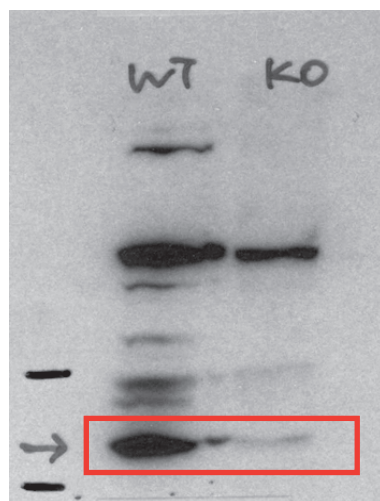

[CEP41]

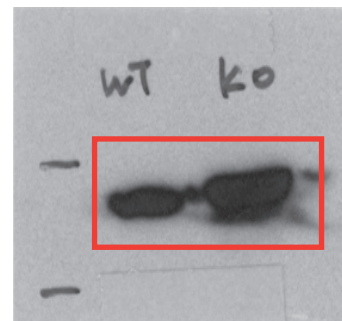

[ACTIN]

Supplement: Supplementary file 7 — Source Data for Appendix [file EMBR-21-e48290-s010.zip › Source_data_App_Fig_S2.pdf]

## Appendix Fig S7B

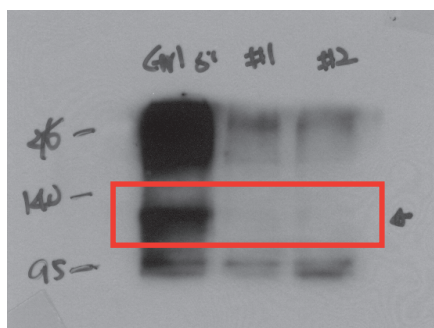

[HIF1 $\alpha$ ]

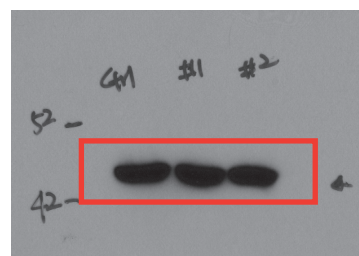

[ACTIN]

Supplement: Supplementary file 7 — Source Data for Appendix [file EMBR-21-e48290-s010.zip › Source_data_App_Fig_S7.pdf]

Figure 6C

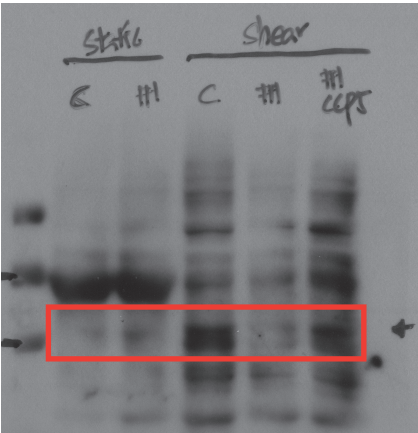

[pAURKA]

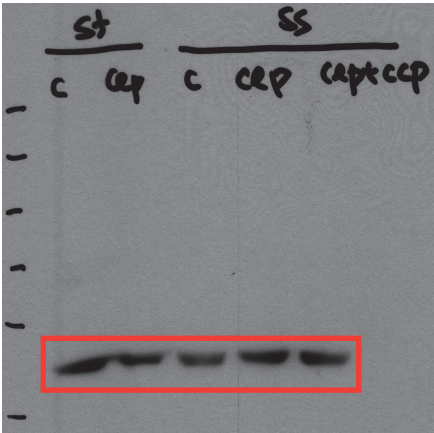

[AURKA]

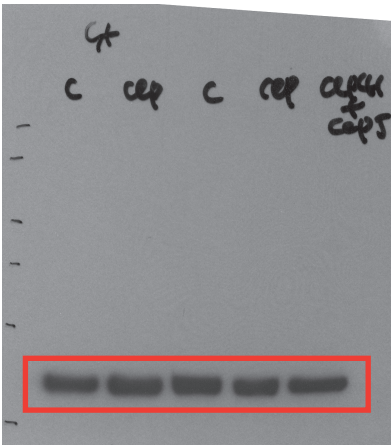

[ACTIN]

Supplement: Supplementary file 9 — Source Data for Figure 6 [file EMBR-21-e48290-s007.zip › Source_data_(6C)-1.pdf]

Figure 6D

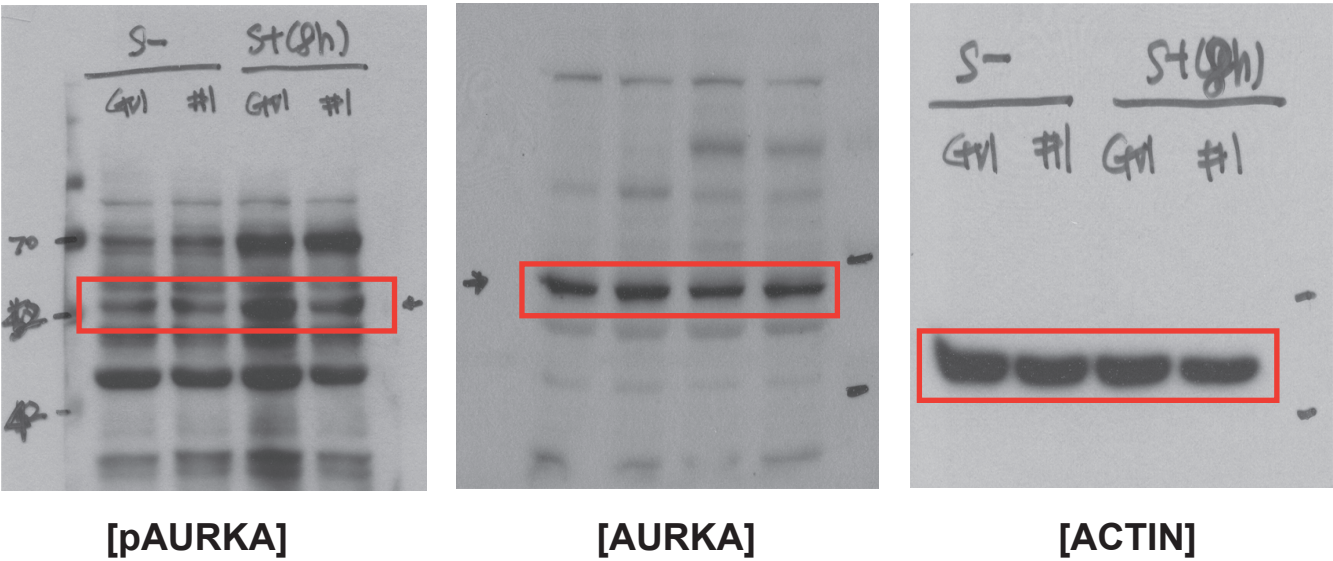

Supplement: Supplementary file 9 — Source Data for Figure 6 [file EMBR-21-e48290-s007.zip › Source_data_(6D).pdf]

Figure 7A

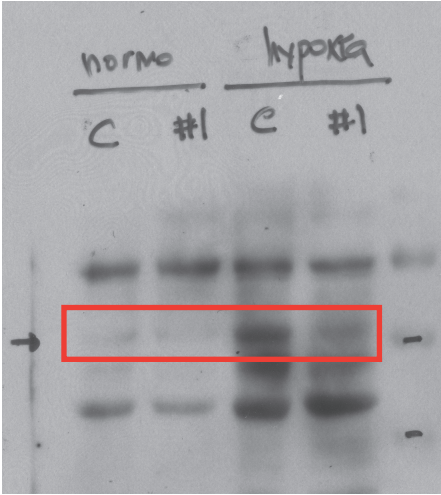

[pAURKA]

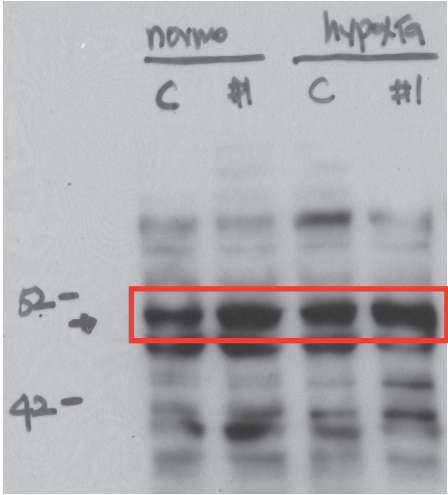

[AURKA]

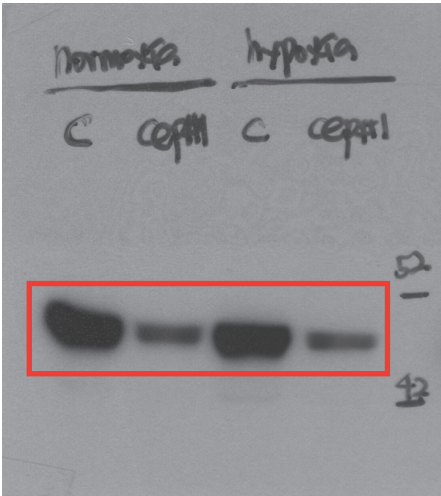

[CEP41]

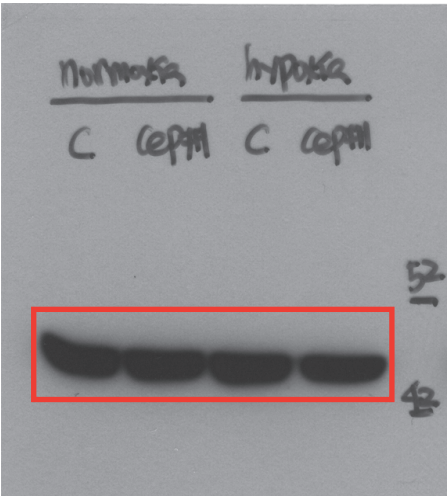

[ACTIN]

Supplement: Supplementary file 10 — Source Data for Figure 7 [file EMBR-21-e48290-s008.pdf]

**Figure 8A**

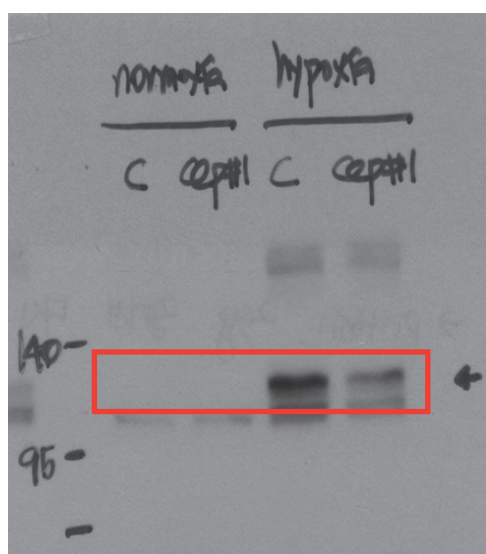

[HIF1 $\alpha$ ]

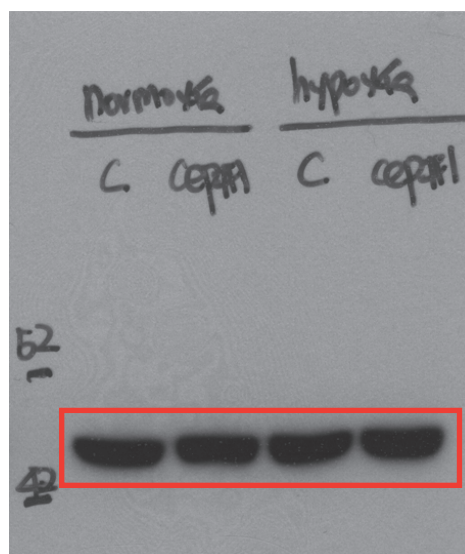

[ACTIN]

Supplement: Supplementary file 11 — Source Data for Figure 8 [file EMBR-21-e48290-s009.zip › Source_data_(8A).pdf]

Figure 8B

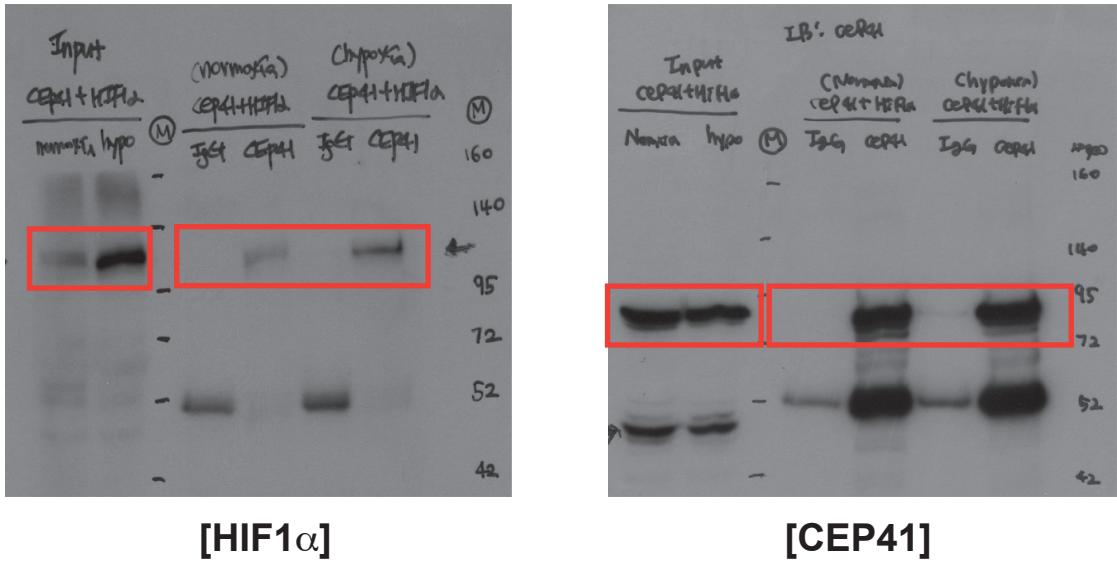

Supplement: Supplementary file 11 — Source Data for Figure 8 [file EMBR-21-e48290-s009.zip › Source_data_(8B).pdf]

Figure 8C

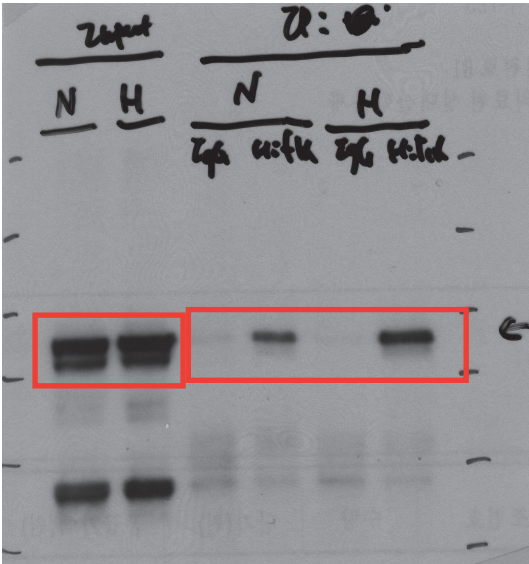

[CEP41]

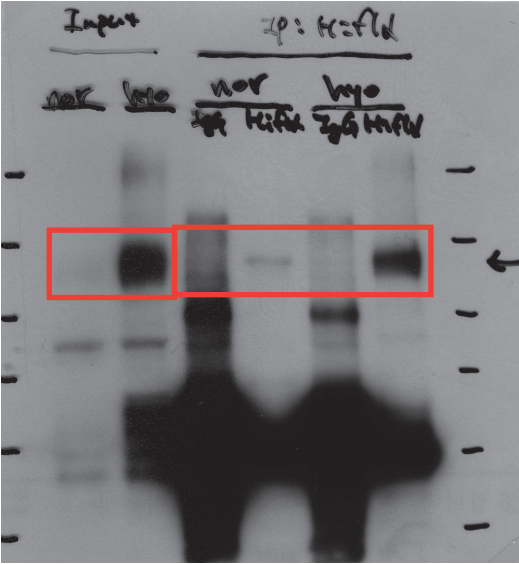

[HIF1α]

Supplement: Supplementary file 11 — Source Data for Figure 8 [file EMBR-21-e48290-s009.zip › Source_data_(8C).pdf]

Figure 8D

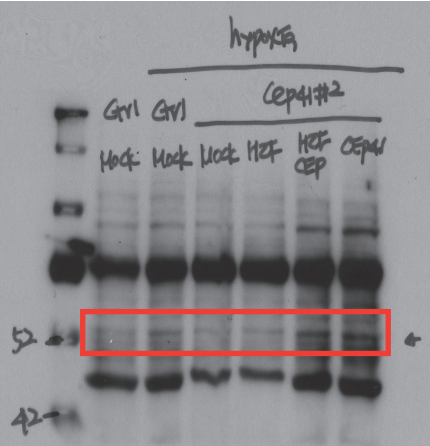

[pAURKA]

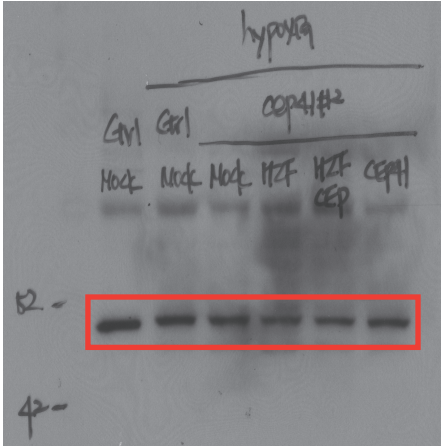

[AURKA]

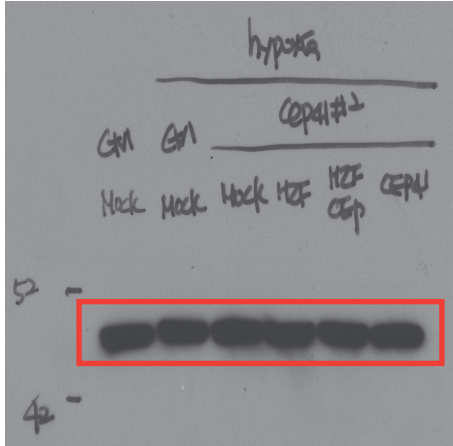

[ACTIN]

Supplement: Supplementary file 11 — Source Data for Figure 8 [file EMBR-21-e48290-s009.zip › Source_data_(8D).pdf]

### Figure 8G

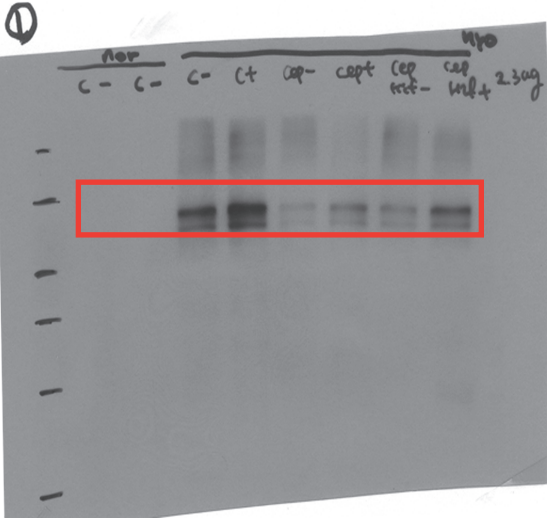

**[HIF1 $\alpha$ ]**

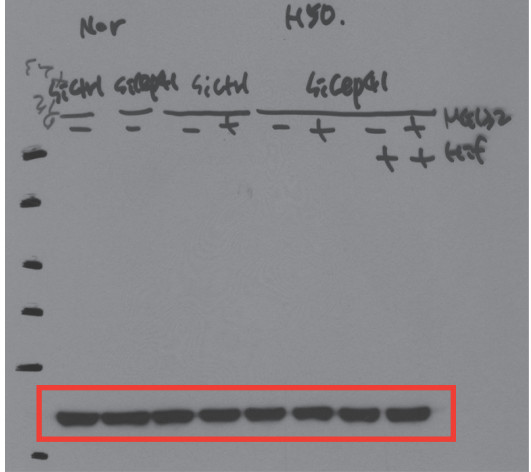

**[ACTIN]**

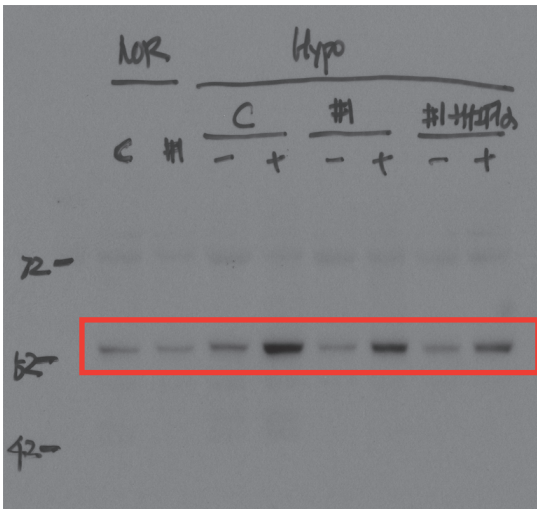

**[pAURKA]**

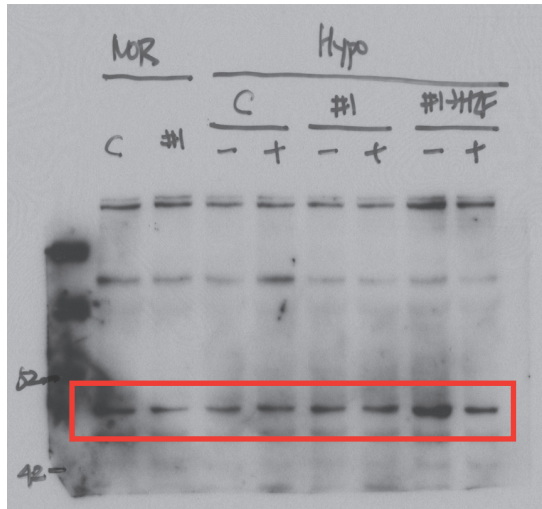

**[AURKA]**

Supplement: Supplementary file 11 — Source Data for Figure 8 [file EMBR-21-e48290-s009.zip › Source_data_(8G).pdf]
